# Optimising Care: Quality Improvement for Sustainable Practices in the Paediatric ADHD Clinic in Wrexham Maelor Hospital

**DOI:** 10.1192/bjo.2024.334

**Published:** 2024-08-01

**Authors:** Catherine Cunning, Mostafa Abdellatif, Qamar Jabeen

**Affiliations:** Betsi Cadwaladr University Health Board, Wrexham, United Kingdom

## Abstract

**Aims:**

To make a case for E-prescribing within the Paediatric Neurodevelopmental Team in Wrexham Maelor Hospital.

To trial a different way of approaching 6 monthly reviews within the ADHD clinic (option for remote reviews).

To show how we could reduce the carbon footprint of the ADHD clinic.

**Methods:**

Process mapping was completed to consider areas in the ADHD prescribing process that could be made more sustainable.

For each patient appointment in the ADHD clinic a questionnaire was completed. The data collection period was over 3 weeks during August and September 2023. Data was collected and interpreted.

**Results:**

99 appointments were offered, 82 appointments attended. 77 appointments were face-to-face and 22 were via telephone. Of the face-to-face appointments, 54 families travelled in by car and 4 used public transport (2 taxis). Of those who commented 31 people found it hard to find parking by the clinic, 13 people did not.

Of the appointments attended face to face via car/taxi (57):

- Average of 4.4 miles travel to the clinic (8.8 miles total journey)
- Shortest journey 1.1 miles (2.2 miles total journey)
- Longest journey 16 miles (32 miles total journey)
- Total patient mileage for these appointments (assuming travel to and from clinic) 855.8 miles

Carbon emissions from the ADHD Clinic:

- Average journey 0.005t CO_2_
- Total journeys 0.472t CO_2_
- Assuming average sized petrol car used
- Extrapolating this data for a whole year approximately: 8.024t CO2 from patient journeys to and from the ADHD clinic

For context the average amount of CO_2_ generated by a single person in the EU is 7.2t.

**Conclusion:**

We have made a case for e-prescribing within the ADHD clinic in Wrexham Maelor Hospital.

The current system impacts on:

- Patient and carer's travel time and convenience.
- Clinician's travel time.
- Carbon emissions.

Alternative processes have the potential to streamline this process making it more sustainable socially, clinically and environmentally.

